# A Case Report of Thoracic Ectopia Cordis in a Hospital in Zanjan, Iran

**DOI:** 10.1155/2024/3912085

**Published:** 2024-09-10

**Authors:** Mehdi Hosseini, Hassan Zamani, Shadi Moghimi, Aref Nekoufar

**Affiliations:** ^1^ Department of Pediatrics Faculty of Medicine Zanjan University of Medical Sciences, Zanjan, Iran; ^2^ Department of Pediatric Cardiology Faculty of Medicine Shahid Beheshti University of Medical Sciences, Tehran, Iran; ^3^ Department of Medicine Faculty of Medicine Zanjan University of Medical Sciences, Zanjan, Iran

## Abstract

**Methods:**

A 17-week-old female neonate with a history of prenatal diagnosis of congenital heart anomaly was admitted to Ayatollah Mousavi Hospital in Zanjan, Iran. For the diagnosis of thoracic ectopia, cordis fetal echocardiography and ultrasonography were performed. Moreover, the noninvasive prenatal testing (NIPT) genetic test performed in the 10th week of pregnancy was evaluated.

**Results:**

The neonate was diagnosed with thoracic ectopia cordis, with the heart located outside the thoracic cavity and covered by a thin membrane. The Z scores of the analyzed maternal venous blood chromosomes were between +6 and −6, and all chromosomes had a low risk in terms of the risk of birth defects. The results of this study revealed that genetic test analysis is not enough to diagnose and predict congenital anomalies and defects. Furthermore, the findings showed that the fetus's mother continued to take folic acid after the first 3 months of pregnancy. This can be one of the risk factors involved in causing this heart defect.

**Conclusion:**

One of the important results of this study was that the diagnostic findings of ultrasound were normal, but the findings of echocardiography were reported as abnormal. This shows that fetal echocardiography is better than ultrasound imaging in diagnosing ectopia cordis. In addition, it is recommended that pregnant women should avoid taking folic acid after the first trimester of pregnancy.

## 1. Introduction

Ectopia cordis is a rare congenital condition in which the heart is located partially or totally outside of the chest cavity. It is estimated to occur in 5.5–7.9 per 1,000,000 live births, with a high mortality rate in the first few days of life. The condition can be classified into four types based on the location of the heart: cervical, thoracic, thoracoabdominal, and abdominal. Thoracic ectopia cordis is the most common type, accounting for approximately 70% of the cases [1, 2].

Thoracic ectopia cordis is a rare congenital condition in which the heart is located outside of the chest cavity. This condition occurs when the chest wall fails to properly develop during fetal development, allowing the heart to protrude through the chest wall. Thoracic ectopia cordis is often associated with other defects in the chest wall, heart, and other organs, making it a complex and serious medical condition [3, 4].

The exact cause of thoracic ectopia cordis is unknown, but it is believed to be linked to genetic factors and environmental influences during fetal development [5]. Symptoms of thoracic ectopia cordis include a visible protrusion of the heart through the chest wall, respiratory distress, cyanosis, and cardiac arrhythmias. The treatment of ectopia cordis usually involves surgical repair to relocate the heart back into the chest cavity and closing the chest wall [6, 7].

International organizations such as the World Health Organization (WHO) and the Centers for Disease Control and Prevention have reported on the rarity and severity of ectopia cordis. Case reports of ectopia cordis have been reported in various countries around the world, highlighting the importance of early detection and appropriate management [8, 9].

There have been several case reports of thoracic ectopia cordis reported in the medical literature worldwide. These cases have highlighted the various presentations of this condition, ranging from isolated ectopia cordis to complex cardiac anomalies. Surgical management has been successful in many cases. However, the prognosis can be variable depending on the severity of the defect and associated anomalies [10, 11].

Surgical intervention is typically required for patients with ectopia cordis, as the condition poses significant risks related to vital function and overall viability. The primary goals of surgical repair include repositioning the heart into the thoracic cavity, closing the thoracic defect, and addressing any associated abnormalities [12, 13]. Various surgical techniques have been developed and refined over time, and the approach can vary depending on the specific type and severity of ectopia cordis, the age of the patient, and the presence of concurrent anomalies [14].

In Iran, there have been limited case reports of thoracic ectopia cordis reported in the medical literature. Goli et al. reported a case report of ectopia cordis with multiple ventricular septal defects and sternal cleft in a newborn. The results of their study showed that the management of ectopy cordis is a challenge for every obstetrician and pediatric surgeon due to its rarity and causes mental distress and anxiety to parents. With early diagnosis, termination of pregnancy can be one of the options [10]. Similarly, Fazea et al. demonstrated the pentalogy of Cantrell associated with ectopia cordis as a case report. The study revealed that the pentalogy of Cantrell should be appropriately evaluated with a multidisciplinary team for effective prenatal counseling and postpartum management, as assessment of infant survival and early diagnosis allow parents to terminate the pregnancy [15].

This highlights the rarity of this condition in the country and the importance of further research to understand the prevalence and outcomes of this anomaly in the Iranian population. The successful surgical management of the case presented in this study adds to the knowledge base on this condition in Iran and emphasizes the importance of early intervention in improving patient outcomes. In this study, we present a rare case report of thoracic ectopia cordis in a hospital in Zanjan, Iran.

## 2. Methods

### 2.1. Case Report

A 2-day-old female neonate with a history of prenatal diagnosis of congenital heart anomaly was admitted to Ayatollah Mousavi Hospital in Zanjan, Iran. Clinical examination revealed the presence of thoracic ectopia cordis, with the heart located outside the thoracic cavity and covered by a thin membrane. Echocardiography and chest radiography confirmed the diagnosis. In addition, the noninvasive prenatal testing (NIPT) genetic test performed in the 10th week of pregnancy showed a low risk for birth defects, with *Z* scores of maternal venous blood chromosomes between +6 and −6.

### 2.2. Case Description

In this case report, a 17-week-old fetus was diagnosed with thoracic ectopia cordis during a routine prenatal ultrasound in a hospital in Zanjan, Iran. The parents were counseled about the condition and the poor prognosis associated with it. Factors that are associated with a poor prognosis include the presence of associated congenital anomalies, the extent of heart exposure, and the presence of cardiovascular compromise. A team of pediatric cardiologists, cardiothoracic surgeons, and neonatologists collaborated in this study.

### 2.3. The Diagnostic Methods

In this study, echocardiography was used to visualize the baby's heart and confirm the diagnosis of ectopy of the thoracic cord. The type of echocardiography device was an echocardiography machine: Vivid GE E95 and also the used probe was Convex C1-5. This test uses sound waves to create an image of the heart and surrounding structures. A chest radiograph was also performed to further assess the position of the heart and other organs in the chest. In addition, noninvasive prenatal genetic testing (NIPT) was evaluated in order to detect congenital anomalies in the 10th week of pregnancy.

### 2.4. The Management of the Patient

The management of the patient with thoracic ectopia cordis was discussed in detail, including postoperative care and follow-up visits. The outcome of the patient was closely monitored, and any complications or adverse events were documented.

### 2.5. Literature Review

The researchers also reviewed the existing literature on thoracic ectopia cordis to provide context and background information for the case report. The findings of the study were summarized and presented in a clear and concise manner, highlighting the importance of early diagnosis and appropriate management of this rare congenital anomaly.

## 3. Results

**Figure 1:**
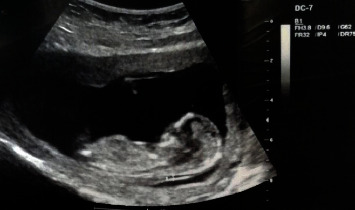
The ultrasound imaging of diagnosed thoracic ectopia cordis.

**Figure 2:**
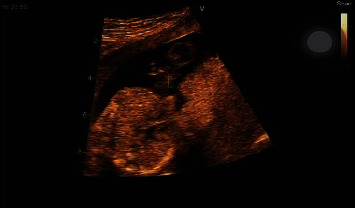
The echocardiographic image of diagnosed thoracic ectopia cordis.

In this case report, the infant was diagnosed with thoracic ectopia cordis using echocardiography, which showed that the heart was covered by a thin membrane. The genetic test of NIPT performed at 10 weeks of gestation showed that all chromosomes were at low risk of birth defects. The *Z* score of maternal venous blood chromosomes was within the normal range, indicating a low risk for chromosomal abnormalities. This case highlights the importance of early screening and prenatal genetic testing in the identification and diagnosis of rare congenital diseases such as thoracic ectopia cordis. The study showed that the *Z* score of the maternal venous blood chromosomes analyzed was between −6 and +6, and all chromosomes were at low risk for birth defects (Table 1). Moreover, the *Z* scores of the sex chromosome aneuploidies were between −2.8 and +2.8, and all chromosomes were at low risk for birth defects (Table 2). In the examined genetic test, there was no high risk of congenital malformation and the *Z*-score of the chromosomes was in the normal range. The results of this study showed that genetic test analysis is not enough to diagnose and predict congenital anomalies and defects. Furthermore, the ultrasound imaging and fetal echocardiographic imaging of diagnosed thoracic ectopia cordis have been depicted in Figures 1 and 2, respectively.

## 4. Discussion

In this study, we report a rare case of thoracic ectopia cordis that presented to a hospital in Zanjan, Iran. The patient was a 6-day-old male infant who was born at term via cesarean section. He presented with a protruding mass in the chest area that was pulsating. Upon examination, it was identified that the heart was located outside the chest cavity, covered by a thin layer of skin. Diagnostic imaging such as echocardiography confirmed the diagnosis of thoracic ectopia cordis. The patient was evaluated by a multidisciplinary team including pediatric surgeons, cardiologists, and neonatologists.

In this case report, echocardiography played a key role in the diagnosis of thoracic ectopia cordis and provided the possibility of planning appropriate interventions. The NIPT also played an important role in this. The NIPT results showed that the risk of chromosomal abnormalities is low and reassured the parents. However, it is important to note that genetic testing alone is not sufficient to diagnose and predict birth defects and abnormalities, as demonstrated in this case.

Goli et al. reported a case report of a 34-week, 6-day-old female fetus with a birth weight of 2040 grams, height of 41 cm, and head circumference of 32 cm. Initial physical examination revealed a responsive neonate with an externalized heart outside the chest, with preservation of the pericardium. In addition, chest wall defects were detected, indicating incomplete septal bone formation. In addition, in this case, the echocardiographic report showed multiple ventricular septal defects. Due to its rarity, the management of ectopia cordis is a challenge for every obstetrician and pediatric surgeon. It causes emotional distress and anxiety for parents. With early diagnosis, termination of pregnancy can be one of the options. When diagnosed late, it requires a multidisciplinary approach and the services of a highly experienced pediatric surgeon to improve prognosis.

In addition, Fazea et al. presented a case report of ectopia cordis and pentalogy of Cantrell in an infant of a healthy 35-year-old woman with no history of fetal toxic exposure or smoking. This baby was diagnosed with this abnormality for the first time in the second trimester of pregnancy and was born by cesarean section in the 35th week of pregnancy. Shortly after birth, he was transferred to the neonatal intensive care unit (NICU) due to progressive respiratory failure, which eventually led to the death of the infant on the second day, along with septicemia. Their study showed that the pentalogy of Cantrell should be appropriately evaluated by a multidisciplinary team for effective prenatal counseling and postpartum management, as assessment of infant survival and early diagnosis allow parents to terminate the pregnancy [15].

Thoracic ectopia cordis is a rare birth defect in which the heart is located outside the chest cavity. This condition occurs when the chest wall does not form properly during fetal development, allowing the heart to protrude through the chest wall [16]. This condition is extremely rare, with only a few hundred cases reported worldwide. The exact prevalence of thoracic ectopia cordis in Iran is not well documented, but it is probably very rare. The management of thoracic ectopia cordis usually involves a multidisciplinary team of healthcare professionals, including cardiologists, cardiac surgeons, and plastic surgeons. Treatment usually involves surgical intervention to repair the chest wall and repositioning the heart in the chest cavity. The success of treatment depends on the severity of the defect and the presence of any associated abnormalities. The prognosis for people with ectopia cordis is usually poor, as the condition is often associated with other serious congenital malformations [17]. However, with advances in surgical techniques and medical care, some people with the disease have been able to survive into adulthood. Long-term management may include ongoing monitoring and medical interventions to resolve any associated complications [18].

Early detection and diagnosis are very important for proper management and treatment [19]. In the case report presented in this study, the ultrasound diagnostic findings were normal, but the echocardiography findings were abnormal. This shows the importance of using fetal echocardiography in the diagnosis of ectopia cordis, as it provides a more accurate and detailed information about the structure and function of the heart. Fetal echocardiography is a specialized ultrasound technique that allows accurate observation of the fetal heart [20]. It is considered the gold standard for diagnosing congenital heart defects because it can detect abnormalities that may not be visible on conventional ultrasound imaging. In this case, abnormal findings on echocardiography would likely alert the medical team to the presence of ectopia cordis and allow further evaluation and planning for delivery and postpartum care. This emphasizes the importance of using appropriate diagnostic tools to diagnose rare and complex congenital conditions such as ectopia cordis. In general, this case report shows the importance of using fetal echocardiography in the diagnosis of ectopia cordis. Early diagnosis and appropriate management are critical to improve outcomes in cases of this rare and challenging condition.

Sepulveda et al. investigated the ultrasound features, antenatal period, and perinatal outcomes in 7 cases of ectopia cordis diagnosed in the first trimester. Four cases were associated with a large omphalocele (pentalogy of Cantrell) and 2 with a body stalk anomaly. In their study, 2 fetuses died in utero, 2 babies died after delivery, and 1 person died at 3 months. The results of their study showed that the diagnosis of ectopia cordis can be easily detected in the first trimester [21].

In the Repondek-Liberska study, conducted between January 1995 and October 1997, eight cases of ectopia cordis were diagnosed prenatally at our institution. Using echocardiography, fetal heart anatomy was categorized as either normal or indicative of a congenital heart defect. In the majority of cases (seven out of eight), additional abnormalities were identified [22]. While some reports have indicated that ectopia cordis can be diagnosed in the first trimester, the average gestational age at diagnosis in this study was 26 weeks. Isolated ectopia cordis is relatively straightforward to diagnose prenatally. Although, managing the consultation with the patient; determining the time, place, and method of delivery; and providing optimal care for the newborn pose more important challenges [23], ectopia cordis is a rare anomaly that pediatricians seldom encounter, even within specialized pediatric cardiology centers. This rarity can pose challenges for sonographers and gynecologists. Nevertheless, pediatric cardiologists should be familiar with the diagnostic algorithm for such cases, particularly when additional abnormalities are present [24].

In the study by Araujo Junior et al., there was a group of 31 fetuses with EC, of which 4 and 27 had partial and complete protrusion of the heart through an abdominal defect in the abdominal thoracic wall, respectively. EC was diagnosed by fetal echocardiography at a mean gestational age of 20.3 ± 8.6 weeks (range: 8–35 weeks). In their study, of the four cases where karyotyping was performed, all had normal results (1–46, XX and 3–46, XY). Five patients showed conotruncal abnormalities and six ventricular septal defects. Termination of pregnancy (TOP) was performed in 15 cases (48%) and 7 pregnant women had spontaneous fetal death (22.5%). Out of the seven fetuses that were born alive, four fetuses died and three babies underwent surgery. Ventricular septal defects and conotruncal malformations were the most common intracardiac defects associated with EC. However, in this group of fetuses with EC, the incidence of PC was lower than that reported in the literature [23].

Furthermore, the findings showed that the fetus's mother continued to take folic acid after the first 3 months of pregnancy. This can be one of the risk factors involved in causing this heart defect. Since hereditary heart diseases were not detected in this woman's family, this defect could be due to folic acid consumption after the first trimester. According to the report of the Food and Drug Administration (FDA), the consumption of 400 micrograms of folic acid (FA) per day is recommended for the prevention of neural tube defects (NTDs) [25]. According to the examination of the medical record of the mother of the fetus, the consumption of folic acid continued after the first trimester of pregnancy in a dose of more than 400 *µ*g/d. Mao et al. reported that pregnant women who did not take folic acid supplements before pregnancy and had a lower dietary intake of folate (vitamin B-9) were approximately 2 times more likely to have congenital heart defects (CHDs) in their children [26]. However, their study was about folic acid intake before pregnancy. Folic acid is critical for DNA synthesis and methylation but excessive folic acid administration may induce the prevalence of DNA point mutations, as reported in the studies of folate deficiency, and these results were observed in Cao's study [27].

Folic acid is an essential nutrient that plays an important role in fetal development, especially in preventing neural tube defects such as spina bifida. It is recommended that pregnant women use folic acid supplements before and during the first trimester of pregnancy to reduce the risk of these defects [28, 29]. However, there is limited evidence on the correlation between folic acid intake after the first trimester of pregnancy and fetal malformations. Some studies have shown that continued folic acid supplementation during pregnancy may still have benefits in reducing the risk of certain birth defects, while others found no significant correlation [30, 31].

## 5. Conclusion

Thoracic ectopia cordis is an extremely rare congenital condition in which the heart is located outside the chest cavity. Our study reports a case of thoracic ectopia cordis in Ayatollah Mousavi Hospital in Zanjan, Iran. Through a careful evaluation of the patient's clinical presentation and imaging studies, we were able to accurately diagnose and manage this rare anomaly. This case report revealed the importance of early screening and prenatal genetic testing in identifying and diagnosing rare congenital diseases such as thoracic ectopia cordis. Echocardiography remains a valuable tool in diagnosing such conditions, while NIPT can provide additional information on the risk of chromosomal abnormalities. The finding also revealed that the diagnostic findings of ultrasound were normal, but the findings of echocardiography were reported as abnormal. This shows that fetal echocardiography is better than ultrasonography in diagnosing ectopia cordis. However, it is important to remember that genetic testing alone is not sufficient to diagnose congenital anomalies, and additional diagnostic procedures may be necessary. In general, a multidisciplinary approach involving different medical specialties is essential for the optimal management of rare congenital conditions such as thoracic ectopia cordis. It is suggested to investigate the correlation between folic acid consumption after the first trimester of pregnancy and the occurrence of ectopia cordis in future studies.

## Figures and Tables

**Table 1:** The noninvasive prenatal genetic testing (NIPT) findings of chromosomes.

Chromosome	*Z* score	Test results	Reference interval
Chromosome 1	0.58	Low risk	−6< Z score <6
Chromosome 2	−0.28	Low risk	−6< Z score <6
Chromosome 3	0.21	Low risk	−6< Z score <6
Chromosome 4	1.15	Low risk	−6< Z score <6
Chromosome 5	0.50	Low risk	−6< Z score <6
Chromosome 6	0.88	Low risk	−6< Z score <6
Chromosome 7	0.26	Low risk	−6< Z score <6
Chromosome 8	−0.72	Low risk	−6< Z score <6
Chromosome 9	−1.72	Low risk	−6< Z score <6
Chromosome 10	0.25	Low risk	−6< Z score <6
Chromosome 11	0.82	Low risk	−6< Z score <6
Chromosome 12	−0.05	Low risk	−6< Z score <6
Chromosome 13	0.29	Low risk	−6< Z score <6
Chromosome 14	−0.46	Low risk	−6< Z score <6
Chromosome 15	−1.72	Low risk	−6< Z score <6
Chromosome 16	−1.26	Low risk	−6< Z score <6
Chromosome 17	−1.98	Low risk	−6< Z score <6
Chromosome 18	0.40	Low risk	−6< Z score <6
Chromosome 19	0.93	Low risk	−6< Z score <6
Chromosome 20	0.98	Low risk	−6< Z score <6
Chromosome 21	−0.39	Low risk	−6< Z score <6
Chromosome 22	1.79	Low risk	−6< Z score <6

**Table 2:** The noninvasive prenatal genetic testing (NIPT) findings of sex chromosome aneuploidies.

Sex chromosome aneuploidies	Z score	Test results	Reference interval
XO	1.29	Low risk	Male −3< Z score <3
XXY/XYY		Low risk	Female −2.8< Z score <2.8
XXX		Low risk	

## Data Availability

The data used to support the findings of this study are available from the corresponding author upon reasonable request.

## Additional Points

*Transparency Statement*. The corresponding author (Shadi Moghimi) affirms that this manuscript is an honest, accurate, and transparent account of the study being reported; that no important aspects of the study have been omitted; and that any discrepancies from the study as planned (and, if relevant, registered) have been explained. All authors have read and approved the final version of the manuscript. The corresponding author (Shadi Moghimi) had full access to all of the data in this study and takes complete responsibility for the integrity of the data and the accuracy of the data analysis.
